# Debonding Detection and Monitoring for CFRP Reinforced Concrete Beams Using Pizeoceramic Sensors

**DOI:** 10.3390/ma12132150

**Published:** 2019-07-04

**Authors:** Shukui Liu, Wei Sun, Hongwen Jing, Zhaoxing Dong

**Affiliations:** 1 State Key Laboratory for Geomechanics and Deep Underground Engineering, China University of Mining and Technology, Xuzhou 221116, China; 2 School of Mechanics and Civil Engineering, China University of Mining and Technology, Xuzhou 221116, China; 3 Key Laboratory of Ministry of Education for Mechanics on Western Disaster and Environment, School of Civil Engineering and Mechanics, Lanzhou University, Lanzhou 730000, China

**Keywords:** CFRP, debonding detection, debonding monitoring, piezoceramic sensor, ultrasonic wave

## Abstract

The bonding status between Carbon Fiber Reinforced Polymer (CFRP) and concrete is one of the key issues for the safety of CFPR-reinforced structures, thus it is of great importance to detect the debonding as early as possible. Instead of detecting the debonding which is artificially set at the very beginning, this paper investigates the feasibility of using low-cost piezoceramic sensors to detect and monitor the debonding of CFRP-reinforced concrete beams in situ. For existing debonding detection, a concrete beam reinforced with CFRP sheet was loaded through the three-point bending test till failure to induce debonding between CFRP sheet and the concrete substrate, and piezoceramic sensors were used to detect the existing debonding by analyzing the receiving ultrasonic waves. In addition, the debonding detection results were further compared with and verified by the vision-based strain testing results. For in-situ debonding monitoring, 10 piezoceramic sensors were used as an array to track the wave transmission changes during the loading process of a CFRP-reinforced concrete beam, and the debonding development process was successfully monitored. The test results show that the low-cost piezoceramic sensors are very effective to generate and receive ultrasonic waves, and are capable of detecting the existing debonding and monitoring of the in-situ debonding process as well.

## 1. Introduction

Over the years, a large number of concrete structures, such as buildings, bridges and tunnels need to be strengthened [1], due to the deterioration caused by loading effects or environmental changes. In many types of strengthening and maintenance methods, the use of Carbon Fiber Reinforced Polymer (CFRP) has increased steadily since 1992 [2,3,4,5,6,7,8,9]. The huge increase of the structures strengthened using externally-bonded CFRP has created new structures that also age and degrade, not only on the CFRP composite and concrete, but also at the interfaces, with consequences for the long-term durability performance of the joint. As a consequence, better knowledge regarding the degradation phenomenon of the adhesively-bonded joints when subjected to different situations is needed, regardless of the causes of degradation [10].

It is known that the durability of CFRP-reinforced concrete structures is often controlled by the adhesive condition between the CFRP sheets and concrete substrate, since the stress shared by the CFRP is transferred from the structures to the CFRP through the interfacial bond. Debonding at the CFRP–concrete interface often results in premature failure of the strengthened structures [11]. Therefore, it is of great importance to detect the existing debonding damage and to monitor the debonding developing process of the CFRP-reinforced concrete structures. However, it is extremely difficult to detect the debonding by a visual inspection, and on-site inspections using bulky devices, such as infrared cameras [12,13], are very time consuming and costly. Thus, low-cost and effective CFRP–concrete debonding detection and monitoring techniques are required.

For concrete-filled steel tubes, Xu et al. [14] used an active interfacial debonding defect detection approach using piezoelectric ceramic transducer (PZT) patch measurements bonded on the outer surface of concrete-filled steel tube members. Sikdar and Ostachowicz [15] proposed an ultrasonic lamb wave-based debonding monitoring technique. While this method was suitable for use on honeycomb sandwich composite structures, Deng [16] also used this technique for detection of composite insulator debonding. Hsieh [17] adopted the impact–echo method to detect debonding flaws at the epoxy–concrete interfaces in near-surface-mounted CFRP strengthening beams. Although most of the flaws could be detected, impact–echo is not suitable for debonding monitoring. Based on free vibration of a thin plate, Xu et al. [18] used an acoustic–optical fiber nondestructive evaluation (NDE) technique for interfacial artificial debonding detection. It is noticed that a lot of the current research is focused on the detection of artificial pre-installed debonding, while debonding caused either by loading or environmental effects might show some different characters. Furthermore, CFRP-reinforced concrete debonding monitoring is also essential to help us get a better understanding of the debonding process and mechanism.

The purpose of this study is to investigate the applicability of using low-cost piezoceramic sensors for CFRP-reinforced concrete debonding detection and monitoring. For debonding detection, a concrete beam reinforced with CFRP sheet was loaded till failure through the three-point bending test to induce debonding between CFRP sheet and the concrete substrate, and piezoceramic sensors were used to detect the existing debonding by analyzing the receiving ultrasonic waves. For comparison purposes, the debonding detection results were also compared with the vision-based strain testing results. For in-situ debonding monitoring, 10 piezoceramic sensors were used as an array to track the wave transmission changes during the loading process of a CFRP-reinforced concrete beam, the debonding developing trend was obtained and the debonding length was also evaluated.

## 2. Materials and Methods 

### 2.1. Test Samples

**Figure 1:**
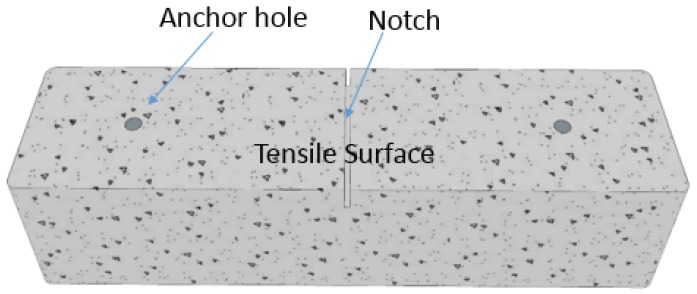
Concrete beam before Carbon Fiber Reinforced Polymer (CFRP) installation.

Two plain concrete beams from the standard test for flexural strength of concrete with dimension of 15.2 cm × 15.2 cm × 61.0 cm (Figure 1) were used as the standard for test samples. The compressive strength was 37 MPa averaged from six cylinder specimens. A notch was cut to initiate the cracking at mid-span, and two holes were drilled for installing CFRP anchors. To preclude failure initiated by flexural cracking, CFRP sheets of 12.7 cm widths were also used to U-wrap the sides of the beams. A gap was made between the two U-wrap CFRP strips to limit the effects of side CFRP strip on flexural contribution [19]. All FRP components including sheets, anchors and U-wraps were made by Tyfo sch-11 up. The nominal tensile modulus, ultimate strain and laminate thickness are 95.8 GPa, 0.0096 and 0.51 mm provided by the manufacturer (Fyfe Co. LLC, San Diego, CA, USA).

After that, the two CFRP-reinforced beams were loaded to failure to generate debonding between CFRP and concrete, one of the beams (namely beam 1 afterwards) were tested after the failure to detect the debonding length, while on the other beam (namely beam 2 afterwards) piezoceramic sensors were pre-installed to monitor the debonding process in situ during loading.

### 2.2. Test Setup of 3-Point Bending

**Figure 2:**
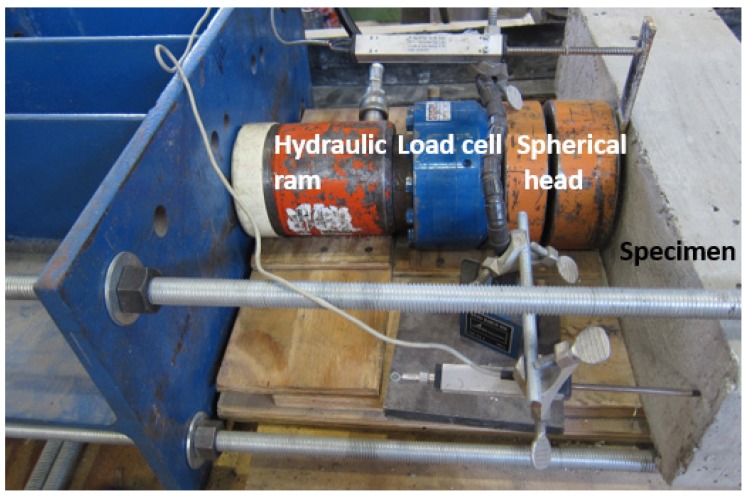
Beam sample loading test setup.

The loading test frame and setup is shown in Figure 2. The testing method followed ASTM C 293 [20], and was modified to carry out the test on the side of the sample so that the vision-based strain testing system could be used [19,21]. The sample was simply supported, with pin and roller support placed 2.5 cm from both ends of the sample. The sample was loaded at mid-span using a hydraulic ram and a 111.25 kN capacity load cell. The load was slowly increased during the test, till the failure of the sample. 

### 2.3. Ultrasonic Measurements

#### 2.3.1. Beam 1 for Debonding Detection after Failure

**Figure 3:**
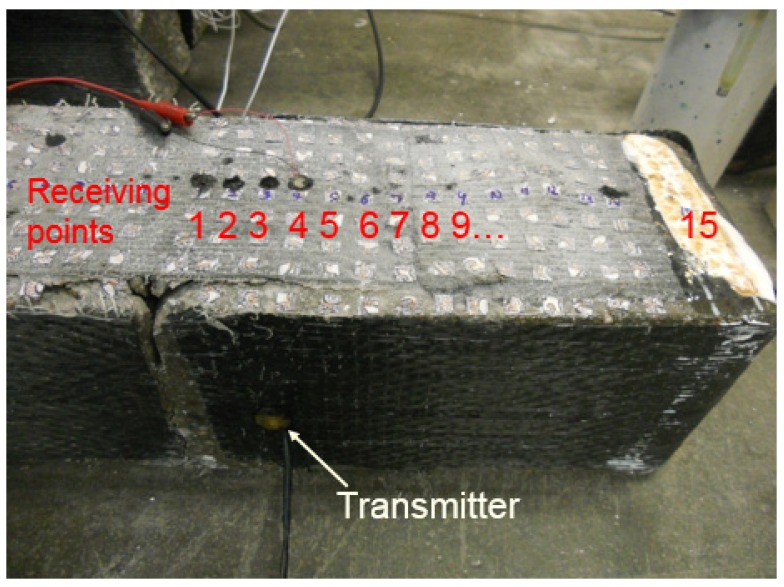
Ultrasonic pitch–catch test setup.

After failure, the right half of the fractured beam was tested and 15 ultrasonic signal receiving points were equally spaced (1.9 cm) from the notch towards the end of the specimen (see Figure 3), with receiving point 1 at 1.3 cm from the notch. Note that the edge of CFRP patch, which is on top of one layer of CFRP sheet and the CFRP anchor fan, is just in between receiving points 7 and 8, and the white points on top of the CFRP the surface were the targets used for strain measurements using the vision system.

In the ultrasonic test, instead of using commercial ultrasonic transducers, low-cost piezoceramic disks (SMD07T02S412) were used to send and receive ultrasonic waves. The piezoceramic actuator was installed on the side surface, while the piezoceramic sensor scanned from receiving points 1 to 15 on the right half of the fractured beam. The actuating sensor was driven by a 100 V, 500 kHz square wave pulse generated from a pulser–receiver (Panametrics 5077PR), and the receiving sensor was connected to the pulser–receiver with a gain of 20 dB. The amplified receiving signals were then digitized by an NI-PXI 5133 digitizer at a sampling rate of 10 MHz, and 10,000 data points were recorded and transferred to a computer. In each measurement, 200 signals were averaged and saved to improve the signal-to-noise ratio.

#### 2.3.2. Beam 2 for Debonding Monitoring during Loading

**Figure 4:**
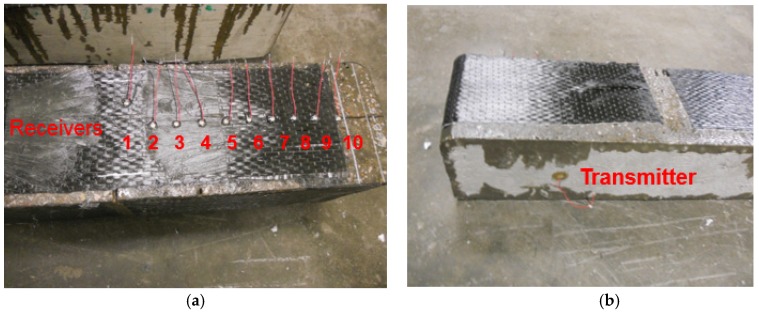
Sensors on beam 2: (**a**) front view (**b**) back view.

Ten receiving piezoceramic disks (Figure 4), with 2.5 cm interval alignment, were mounted on the CFRP surface using epoxy, with sensor 1 at 1.3 cm from the notch. One piezoceramic disk was mounted on the opposite concrete surface of the beam to serve as an actuator. The permanently installed sensors give more repeatable signals than moving the sensor for scanning. 

The beam was loaded monotonically at 4.45 kN intervals. The beam was monitored till 57.85 kN of loading, after which the beam was loaded continuously till failure. Nondestructive testing (NDT) data was acquired at the loading intervals. The data acquisition procedure was mostly the same as used in Section 2.3.1 except an Agilent 34903 module switch was used to scan receiving sensors in sequence.

## 3. Results and Discussion

### 3.1. Debonding Detecion after Failure

**Figure 5:**
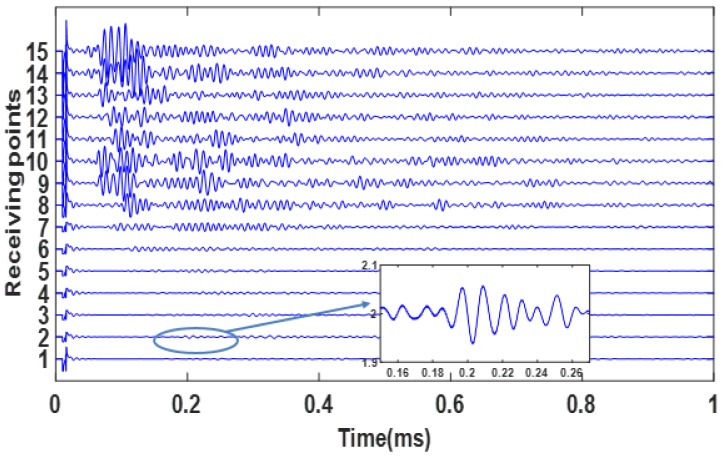
Time domain signals obtained from different receiving points on beam 1 after failure.

Debonding between CFRP sheet and concrete substrate will significantly decrease the energy transmission between the sensors. For beam 1, time domain signals from receiving point 1 to 15 are shown in Figure 5. 

**Figure 6:**
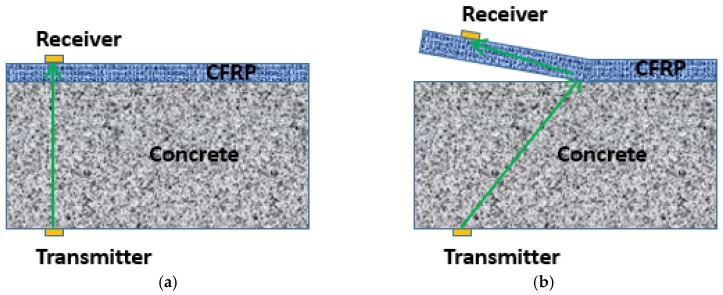
Wave transmission: (**a**) before debonding (**b**) after debonding.

It is clearly seen that before receiving point 7 (12.7 cm from the notch), the amplitudes of the signals are considerably low, which implies that from receiving points 1 to 7, the CFRP was debonded. Note that the edge of the anchor patch was just in between receiving points 7 and 8 (see Figure 6). This result shows that the debonding approximately developed to the edge of the anchor patch. Thus the estimated debonding length in this case is around 12.7 cm.

Though the signal amplitudes of receiving points 1 to 7 are considerably low, signals still could be obtained if a closer look was taken (see the subplot in Figure 5). The reasons for this are demonstrated in Figure 6. As seen in the figure, the wave could directly propagate through the concrete and CFRP bonding surface with high amplitude before debonding happens, while a folded wave path is formed when debonding happens and lower ultrasonic energy is transmitted. 

**Figure 7:**
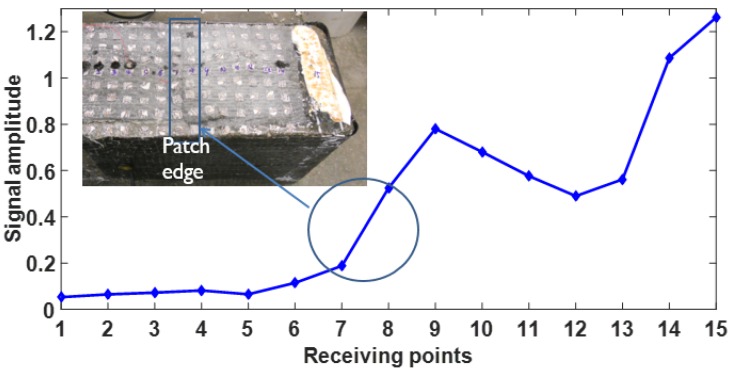
Signal amplitudes at different measuring points.

Figure 7 shows the signal amplitudes of different receiving points. It is noticed that the signal amplitudes of receiving points 1 to 7 are less than 20% of the amplitude at receiving point 15, under which there is no CFRP sheet. This huge difference implies that the energy transmission could be used as an effective index to evaluate the debonding between CFRP sheet and concrete substrate. Amplitude variations were observed of the signals obtained from receiving points 8 to 15. This is probably caused by the layout of CFRP installations. Under receiving points 1 to 7, only one layer of CFRP sheet was installed and the CFRP surface was even, while under receiving points 8 to 14, the CFRP was installed in the order of CFRP sheet, CFRP anchor fan and CFRP patch, and the surface was uneven due to the slope of the anchor fan. Notably, the anchor hole is under receiving point 12, and at this point the thickness of CFRP achieves the peak, due to the higher attenuation of CFRP, a trough was observed in Figure 7. In contrast, the signal amplitude at receiving point 15 is the largest because under the receiving point there is no CFRP, and receiving point 15 was directly put on top of the concrete surface and the signal was attenuated the least.

**Figure 8:**
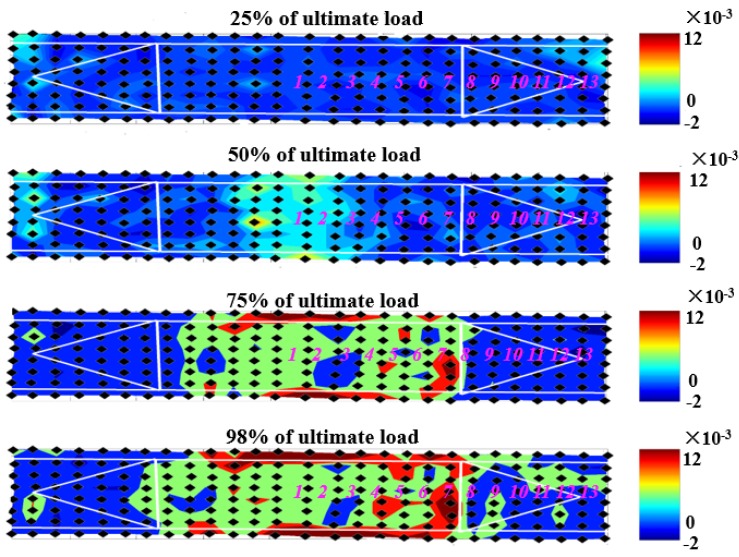
Principal strain at different loading levels.

In addition, the nondestructive debonding test results were further compared with the strain measurement results from the vision-based strain testing system [19]. The vision-based strain testing system is based on digital image correlation and was used to monitor the CFRP surface strain field change during loading. Four loading stages of strain contour were listed in Figure 8, the anchor fans were outlined using white solid lines, and the ultrasonic wave receiving points were also covered on the figure for comparison purposes (note that ultrasonic tests were performed after the beam failure). It could be seen that at 25% of ultimate load, nearly uniform small strains occurred across the CFRP strip, while at 50% of ultimate load, strains developed near mid-span with an average value of 0.0057, which is larger than 0.004, and this is corresponds to an upper-bound debonding strain as defined by ACI 440 [22]. The CFRP strips were likely debonded from the concrete substrate at mid-span at this load level. At 75% and 98% (100% means failure, and images at this stage could not be used due to the huge movements of the sample at failure) of ultimate load, strains were relatively uniform across the CFRP surface between the two anchors, indicating that the strip between the anchors had likely totally debonded from the concrete substrate, and the maximum strain at 98% ultimate load level is around 0.013. It is noticed that when the load approached 98% of the ultimate load, on the right part of the beam, the principal stain distribution area approximately corresponded to the area stretched from ultrasonic wave receiving points 1 to 7, and a clear edge could be seen in the strain contour between receiving points 7 (higher strain area) and 8 (lower strain area). This result agrees very well with the nondestructive test results shown in Figure 7. In Figure 7 signal amplitudes begin to increase rapidly right after receiving point 7. This implies that the ultrasonic method could be used to detect the debonding between CFRP sheet and concrete substrate effectively.

### 3.2. Debonding Monitoring during Loading

As for debonding monitoring on beam 2, ultrasonic signals of sensors 1 to 10 (23.6 cm from the notch) were obtained at different loading levels, and signal amplitudes were used to describe the debonding process.

**Figure 9:**
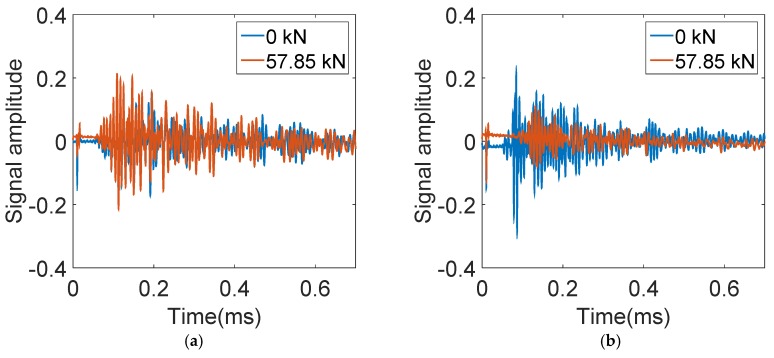
Signals of (**a**) sensor 9 and (**b**) sensor 2 obtained at 0 kN and 57.85 kN.

Figure 9a shows the signals of sensor 9 (21.3 cm from the notch) obtained at 0 kN and 57.85 kN of loads, respectively. It is noticed that the early parts of the two signals are almost identical: the peak amplitude and the first arrival time are almost the same. This implies that, at 57.85 kN, the CFRP at sensor 9 is still well-bonded with the concrete substrate. This is reasonable since sensor 9 is far away from the notch in the center of the beam.

For comparison, signals of sensor 2, which is 3.8 cm from the notch, obtained at 0 kN and 57.85 kN of loads are shown in Figure 9b. It is clearly seen that the amplitude at 57.85 kN is considerably lower than when the beam was intact (0 kN). In addition, the first arrival time at 57.85 kN is also delayed. This implies that at 57.85 kN, the CFRP at sensor 2 is totally debonded with the concrete substrate.

**Figure 10:**
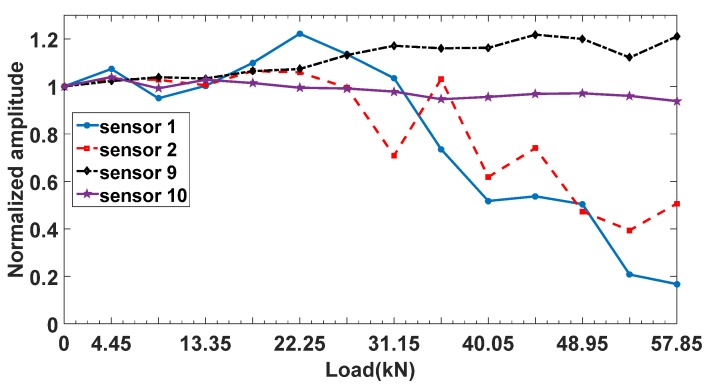
Debonding process of the beam.

In order to get a better understanding of the debonding process, normalized signal amplitudes obtained from sensor 1 (1.3 cm from the notch), 2, 9 and 10 were shown in Figure 10. It is seen that debonding first occurred at sensor 1 at around 31.15 kN of loading, and then propagated to sensor 2 at 35.60 kN of loading. The CFRP at sensor 9 and 10 remained well-bonded during the whole loading process.

**Figure 11:**
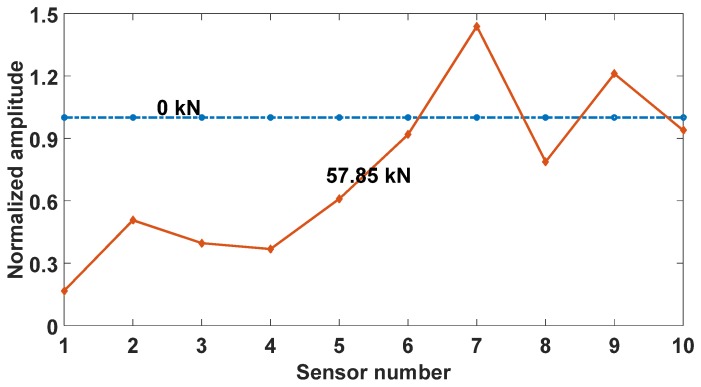
Debonding length evaluation.

The CFRP debonding length evaluation at 57.85 kN of loading was given a try in Figure 11. As seen in the figure, the amplitudes of sensors 1 to sensor 4 at 57.85 kN of loading are around 40% of the original amplitude (at 0 kN). Although an amplitude increase trend was observed from sensors 4 to sensor 6, the normalized amplitude is still well below 1.0. This implies that the area from sensors 4 to sensor 6 probably corresponding to a partially debonding zone.

## 4. Conclusions

This study has investigated the feasibility of using low-cost piezoceramic sensors to detect and monitor the debonding of CFRP-reinforced concrete beams in situ. The following conclusions could be drawn:

(1) In the debonding detection test, the signal amplitude obtained in the debonding area is less than 20% of the amplitude obtained from the sensor attached to the concrete surface, and a 12.7 cm debonding length was estimated. The results were compared and verified by the strain measurement results from the vision-based strain testing system. The ultrasonic pitch–catch method using piezoceramic sensors showed feasibilities for locating the existing debonding between CFRP sheet and concrete substrate.
(2) In the debonding monitoring test, at 57.85 kN of load, the amplitudes of sensors 1 to 4 were 40% less than the amplitudes before loaded. Other than this amplitude drop, a first arrival delay phenomenon was also observed. The debonding process of CFRP-reinforced concrete beam during loading could be successfully monitored by piezoceramic sensors in situ.
